# Corneal Perforation during Combination Chemotherapy including Cetuximab in a Patient with a History of Herpetic Keratitis

**DOI:** 10.1155/2020/6802408

**Published:** 2020-07-07

**Authors:** Keiichi Aomatsu, Koji Sugioka, Aya Kodama-Takahashi, Masahiko Fukuda, Hiroshi Mishima, Shunji Kusaka

**Affiliations:** ^1^Department of Ophthalmology, Nara Hospital, Kindai University Faculty of Medicine, Nara, Japan; ^2^Department of Ophthalmology, Kindai University Faculty of Medicine, Osaka, Japan

## Abstract

**Purpose:**

To report a case of corneal perforation, in a patient with a history of herpetic keratitis, during combination chemotherapy including cetuximab.

**Case:**

We report the case of a 71-year-old man who was diagnosed with a hypopharyngeal carcinoma and received radiation therapy combined with cetuximab, the epidermal growth factor receptor (EGFR) inhibitor monoclonal antibody. He was referred to us because of ocular hyperemia and corneal perforation in his left eye. In spite of conservative therapy, his corneal perforation was exacerbated, with iris incarceration into the wound site and exposure to the surface of the cornea. He therefore discontinued treatment with the combination chemotherapy and underwent lamellar keratoplasty using a preserved donor cornea. After treatment with cetuximab resumed, there was no recurrence of the corneal perforation.

**Conclusion:**

We have presented the first case of cetuximab-related corneal perforation in a patient who had a history of recurrent herpetic keratitis. EGFR inhibitors, such as cetuximab, can induce corneal perforation in cases with a history of herpetic stromal keratitis.

## 1. Introduction

Cetuximab is a molecular-target drug that directly inhibits epidermal growth factor receptor (EGFR), thereby suppressing tumor cell proliferation and acting as an antitumor therapy. In recent years, the combination use of platinum-containing drugs and fluorouracil with cetuximab has improved the survival rate of patients with recurrent and/or metastatic head and neck squamous cell carcinoma. In Japan, cetuximab can be applied in cases of unresectable, progressive, and/or recurrent EGFR-positive colorectal cancer or in head and neck cancer. Associated with its EGFR-inhibition effect, side effects of cetuximab, including corneal disorders, have been reported. These disorders include persistent corneal erosion [1] and filamentary keratitis [2]. Both types of corneal disorders were ameliorated through conservative therapy or discontinuation of the suspect drug. In this report, we present a case of corneal perforation, likely caused by cetuximab, which required surgical treatment because it was difficult to achieve improvement with conservative medical treatment. The present patient developed a corneal perforation after conventional chemotherapy, as well as cetuximab treatment for the previous 6 months, for the treatment of a hypopharyngeal carcinoma with cervical lymph node metastases. As mentioned above, a few cases of cetuximab-related corneal disorders have been reported before now, but there have not been any reports of corneal perforation; this case is the first such report, as far as we know.

## 2. Case Presentation

**Figure 1:**
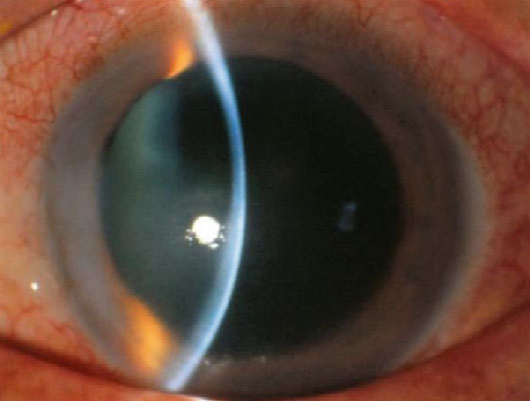
Slit-lamp photographs of the patient's left eye. Six years before the first visit, a portion of his cornea became slightly thin and developed a scar in the paracentral cornea after an episode of herpes simplex keratitis.

A 71-year-old man was referred to the authors because of ocular hyperemia and corneal perforation in his left eye. He had been under regular observation by his ophthalmologist because of recurrent herpes simplex keratitis in his left eye from 8 years before the first visit.  Figure 1 shows a slit-lamp photograph of the patient's left eye 6 years before the first visit. The nasal portion of his left cornea became slightly thinner than normal, and a scar was left in the paracentral cornea after an episode of herpes simplex keratitis. Until about 1 year before the first visit, however, his best-corrected visual acuity in the left eye remained at 20/20. Conversely, no pathological changes were observed in the patient's right cornea. Later, he was diagnosed with a hypopharyngeal carcinoma (stage IVA) with cervical lymph node metastases, so he underwent chemotherapy (5-fluorouracil (5-FU), docetaxel) starting from 8 months before the first visit. Six months before the first visit, he underwent an operation for cervical lymph node dissection. He received radiation therapy combined with 7 cycles of cetuximab (EGFR inhibitor monoclonal antibody) treatment (400 mg/m^2^) for 7 weeks after the operation. Two months before the first visit, computed tomography (CT) revealed the recurrence of cervical lymph node metastases and the appearance of supraclavicular lymph node metastases. He was then additionally treated with two cycles of cetuximab combined with carboplatin and 5-FU before he was referred to us. His medical history was significant for chronic obstructive pulmonary disease.

**Figure 2:**
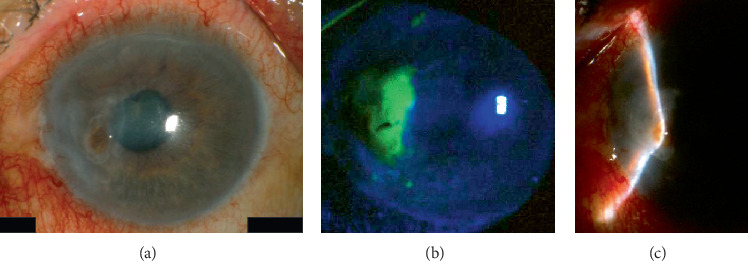
Slit-lamp photographs of the patient's left eye at his initial visit. Slit-lamp examination revealed corneal perforation (a). Corneal epithelial defects were seen using fluorescein staining (b). The space in his left anterior chamber had almost disappeared (c).

**Figure 3:**
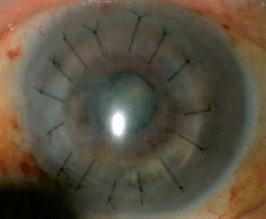
Slit-lamp microscopy image of the patient's left eye, taken on the day after his operation. The corneal graft was clear, and the depth of the anterior chamber was nearly normal.

At the patient's initial visit with us, slit-lamp examination revealed a corneal perforation with a corneal epithelial defect in his left eye. In addition, his left anterior chamber space had almost disappeared (Figures 2(a)–2(c)). We treated him with a medical-bandage soft contact lens and topical antibiotics, such as levofloxacin eye drops (Cravit, Santen, Osaka, Japan) and ofloxacin eye ointment (Tarivid, Santen, Osaka, Japan). However, 2 days later, his corneal perforation was exacerbated, with iris incarceration into the wound site and exposure to the surface of the cornea. We therefore consulted the patient's medical oncologist. Eventually, chemotherapy of cetuximab combined with carboplatin and 5-FU was stopped by the medical oncologist. Two days later, we carried out lamellar keratoplasty using a preserved cornea after hospital admission. The host cornea, including the perforated area, was trephined with a 7.0 mm diameter, and the stromal lamellae were removed from Descemet's membrane during the operation. A graft, 0.5 mm larger in diameter than the recipient bed, and resected Descemet's membrane and endothelium were transplanted using 10-0 nylon sutures. The slit-lamp microscopy image from the day after the operation is shown in Figure 3. The corneal graft was clear, and the anterior chamber was almost back to normal depth. The patient left our hospital 4 days after the operation. From 1 week to 1 month after the operation, he suffered from a graft-stitch abscess accompanied by anterior chamber hypopyon; he received antibiotics topically and systematically, and the corneal infection improved to some extent. During the break from chemotherapy, CT revealed increased cervical lymph node swelling and recurrence of hilar lymphadenopathy and mediastinal lymphadenopathy. One month after the eye operation, treatment with cetuximab, in combination with carboplatin and 5-FU, resumed for a third cycle. After retreatment with cetuximab therapy, there was no recurrence of the corneal perforation but the patient died from progression of the tumor invasion and metastases about 5 months after the eye operation.

## 3. Discussion

In this case, the patient was diagnosed with hypopharyngeal carcinoma with cervical lymph node metastases. He was treated with cetuximab, a so-called molecular-target drug, in addition to conventional chemotherapy for about 6 months. After that, his left cornea developed a corneal perforation. A few cases of cetuximab-related corneal disorders have been reported before now [1, 2], but there have not been any reports of corneal perforation; this case is the first such report, as far as we know. Although medical treatments including oral doxycycline are effective in the management of corneal melting [3], we chose surgical treatment because corneal perforation rapidly progressed in the present case.

EGFR is expressed in corneal epithelial cells and plays a crucial role in corneal wound healing and homeostasis. In animal experiments, EGF mRNA was significantly increased in rabbit lacrimal gland tissue after corneal epithelial injury [4], EGFR was phosphorylated on tyrosine residues after injury, and the phosphorylation levels increased [5]. In a rat model, epithelial wound healing was significantly, and dose-dependently, delayed following systemic administration of an EGFR tyrosine kinase inhibitor [6].

The first class of EGFR inhibitors, which specifically and competitively target EGFR, thereby inhibiting it in extracellular receptors, includes monoclonal antibodies such as cetuximab and panitumumab. The second class of EGFR inhibitors consists of intracellular tyrosine kinase inhibitors, such as erlotinib and gefitinib. Among EGFR inhibitors other than cetuximab, a case of corneal perforation caused by panitumumab treatment, which required penetrating keratoplasty, has been reported [7]. Of reports of corneal perforations caused by erlotinib treatment, there was one case requiring lamellar keratoplasty [7] and another case in which the perforation self-healed following temporary discontinuation of erlotinib treatment [8]. Thus, including our case, several reports of corneal perforation associated with the use of the two types of EGFR inhibitors have emerged in recent years; these findings suggest that physicians should exercise caution in using these drugs.

In the present case, in addition to cetuximab, conventional anticancer drugs, including 5-FU, docetaxel, and carboplatin, also had been administered before the occurrence of the corneal perforation. To investigate the relationship between corneal perforation and these other drugs, we searched the published literature. There have been no reports of corneal disorders related to carboplatin. Neither a corneal stromal infiltration nor any persistent fibrotic changes that might seriously affect vision have ever been reported in patients treated with docetaxel [9]. There was one case of postoperative sterile corneal ulceration and corneal perforation after glaucoma filtering surgery that used 5-FU [10]. However, there have been no reports of corneal perforation related to systemic administration of 5-FU.

There have been reports of different kinds of ocular surface disorders caused by EGFR inhibitors, including cetuximab, panitumumab, and erlotinib. In those reports, dry eye syndrome [7], insertion of punctual plugs for dry eye [8], and prior LASIK [11] were listed as prior ocular surface disorders in some patients, whereas no ocular history was reported in other cases. In our case, the patient had a past ocular history of herpetic stromal keratitis. Therefore, we could not discount the possibility of a recurrence of herpetic keratitis at the initial visit. In fact, herpetic stromal keratitis can become chronic and recurrent, and severe inflammation may lead to thinning and perforation within a short period of time. However, in our case, pathognomonic corneal epithelial signs, such as dendritic ulcer or stromal infiltration or edema, could not be identified. Moreover, part of the patient's left cornea had become thinner because of recurrent herpetic stromal keratitis before the initial visit; the perforation occurred in the thinned area. It is difficult to elucidate the mechanism of activation of herpetic keratitis by anti-EGFR antibodies, because there is no study to date about a cause-and-effect relationship between anti-EGFR antibody administration and the activation of herpes. Therefore, the patient was diagnosed with a corneal perforation potentially caused as a side effect of cetuximab treatment, and we advised his medical oncologist to halt the cetuximab treatment at once. The precise pathogenesis of the disease in this case remains unclear. However, it can be speculated that EGF signal suppression, by the EGFR inhibitor, contributed to a deficiency in corneal wound healing and that, consequently, the chronic corneal epithelial defect induced degeneration of stromal collagen leading to a perforation in the thin, scarred area of the cornea.

In conclusion, we have presented the first case of cetuximab-related corneal perforation in a patient who had a history of herpetic stromal keratitis. When a patient is treated with an EGFR inhibitor such as cetuximab, their medical oncologist and ophthalmologist should be aware of the possibility of corneal perforation via corneal wound-healing deficiency, especially in cases with a history of a corneal disorder.
